# Does the unconditional cash transfer program in South Africa provide support for women after child birth? Barriers to accessing the child support grant among women in informal work in Durban, South Africa

**DOI:** 10.1186/s12889-022-12503-7

**Published:** 2022-01-16

**Authors:** Silondile Luthuli, Lyn Haskins, Sphindile Mapumulo, Christiane Horwood

**Affiliations:** Centre for Rural Health, University of KwaZulu-Natal, George Campbell Building, Howard College, Durban, South Africa

**Keywords:** Cash transfer, Child support grant, Informal work, Maternal health, Child health, Child development, South Africa, Africa

## Abstract

**Background:**

The child support grant (CSG) is the largest unconditional cash transfer program in Africa and aims to alleviate poverty and improve child health and nutrition in low-income families in South Africa. Among informal working women, the CSG is an important source of income after childbirth when informal workers are unable to work, but reports suggest that women experience delays in accessing the CSG. We explore experiences and challenges of accessing the CSG among informal workers in Durban, South Africa.

**Methods:**

We undertook a longitudinal mixed-methods cohort study. Women informal workers were recruited during pregnancy and followed-up for up to one year after the baby was born. Quantitative questionnaires and semi-structured in-depth interviews were used to collect data about women’s plans for applying for the CSG, the application process, use of the CSG in the household, and household food insecurity. Interviews were conducted in IsiZulu by experienced researchers. Descriptive analysis of quantitative data used SPSS v26, and framework analysis using NVIVO v12.3 was used for qualitative analysis.

**Results:**

Twenty-four informal working women were enrolled. The CSG received for older children was reported as an important and reliable source of income for mothers after childbirth. However, delays receiving the CSG for the new baby meant this support was unavailable to first-time mothers. The complex application process for the CSG required mothers to travel to various government departments to complete the required documentation, often taking the baby with them. This was costly and time-consuming for mothers who were already vulnerable, and led to delays in obtaining CSG funds. Many women experienced moderate or severe food insecurity before and after the baby was born. As a result, some mothers had to return to work earlier than planned, disrupting childcare and breastfeeding.

**Conclusions:**

Cash transfer programmes can effectively support low income households and improve outcomes for mothers and children. In South Africa there is a need for innovative approaches to streamline CSG applications, so women can access the funds immediately post-delivery to fill a resource gap and provide support at a vulnerable time for mothers and their children.

## Background

Cash transfer (CT) programmes are a social protection strategy that has been adopted in increasing numbers of low and middle-income countries, to provide income and alleviate poverty in low income families and communities [1, 2]. The key aim of CTs is to break the cycle of intergenerational poverty and invest in the human capital of poor families [2]. Cash transfers may be unconditional or have conditions attached, for example school attendance, antenatal clinic attendance or facility-based delivery [2–5]. Cash transfers have been shown to successfully improve household poverty, health, nutrition and education indicators [1, 6–8], but have so far failed to convincingly address gender inequalities [2, 9]. CTs can be aimed at a variety of beneficiaries depending on the objectives of the programme, including school-going children, pregnant women, rural families and carers of children [10–12].

The Child Support Grant (CSG) in South Africa (SA) is one of the largest unconditional CTs in Africa, with over twelve million beneficiaries [13]. The CSG was established in 1998 with the aim of alleviating child poverty and improving health and development outcomes for children in poor communities [14, 15]. This aim was later expanded to include improvement in school attendance among children who receive the CSG [16]. The grant is provided to mothers or child carers in low-income families, and provides support to children from birth until 18 years. Studies on the CSG over almost two decades have consistently demonstrated the benefits of the grant on child nutrition, development and school attendance [17, 18].

Food insecurity is common in SA, with an estimated 25% of the population living below the food poverty line and considered food insecure [19]. A high proportion of female-headed households means that women frequently take the primary responsibility as breadwinners, providing food and basic shelter for their children [20, 21]. Child-bearing can have the effect of marginalising women, both by reducing income-earning capacity and increasing their financial burdens [22]. Almost all grant recipients are women [23], so the grant has the potential to empower women to care for their children [21]; and the CSG is an important source, sometimes the only source, of income for women in poor communities [15]. The CSG often has multiple uses within the household [24], but evidence suggests that a high proportion of the money is spent on food [18, 24], and receipt of the grant is associated with improved food consumption and dietary diversity among children [16, 21, 23] and improved household food security [25]. However, a recent review suggests that although food insecurity and hunger has improved among recipients, the grant is insufficient to entirely prevent food insecurity and malnutrition [25]. Further, there has been little improvement in child nutrition since introduction of the CSG, with continued high rates of stunting among South African children [25].

One-third of women workers in South Africa are informal workers. These are a vulnerable group with low paid, insecure jobs, no access to maternity protection, and high rates of food insecurity [26]. Studies among informally working women suggest that the CSG is an important additional source of income, with many informally working women relying on the CSG received for their older children for support immediately after childbirth [26–28]. As heads of households, women carry responsibilities for feeding and childcare, which has a negative impact on their ability to work and support their family.

There is substantial evidence since the inception of the CSG that women face challenges with accessing the CSG [14, 17, 18, 23, 29]. In this paper, we focus on barriers to accessing the CSG among informally working women in Durban, South Africa and the effect this has on food security and vulnerability after childbirth.

## Methods

The data presented in this paper forms part of a larger longitudinal mixed methods cohort study that explored the relationship between childcare and informal work [27, 30]. We followed-up pregnant informal workers until they returned to work or the child reached the age of one year. A longitudinal research design allowed researchers to explore participants’ lived experiences as they changed over time. Quantitative and qualitative data were collected through a series of interviews to explore experiences of applying and accessing the CSG funds and to determine household food insecurity at different time points.

### Study setting

The study was conducted in two townships in Durban, KwaZulu-Natal (KZN). The Durban area is characterized by high rates of unemployment, poverty, and low income in the population [31]. There is a high proportion of female-headed households within the two townships, approximately 47% and 38% [31]. Mothers and primary caregivers of children from low-income households are eligible to apply for the CSG of R420 (approx US$30) which is paid monthly for each child from birth until the age of 18. Requirements for the CSG application are shown in Table 3.

### Participants and Sampling

A sample size of 20 participants was estimated as enough to reach data saturation, based on our previous experience conducting qualitative research [32, 33]. Twenty-four informal working women were enrolled in the cohort to allow for loss to follow-up over the extended period of follow up. For the purposes of the study informal workers were defined as workers who did not have a work contract, and were not contributing to the South Africa mandatory Unemployment Insurance Fund (UIF). Domestic workers were separately defined as women working in private households undertaking domestic and childcare work, regardless of whether they had a contract or paid UIF.

Participants were recruited in the antenatal clinics at two primary health care clinics in the Durban area during their last trimester of pregnancy. Eligible participants were aged 18years or older, between 32 and 38 weeks pregnant, and were designated as informal workers according to the study definition. Women were excluded if they worked less than three days a week or had been in informal work for less than 6 months or were planning to leave the area before the child was 6 months old. All eligible participants were requested to participate and underwent a three-stage recruitment process before being formally enrolled in the cohort.

### Data collection

**Fig. 1:**
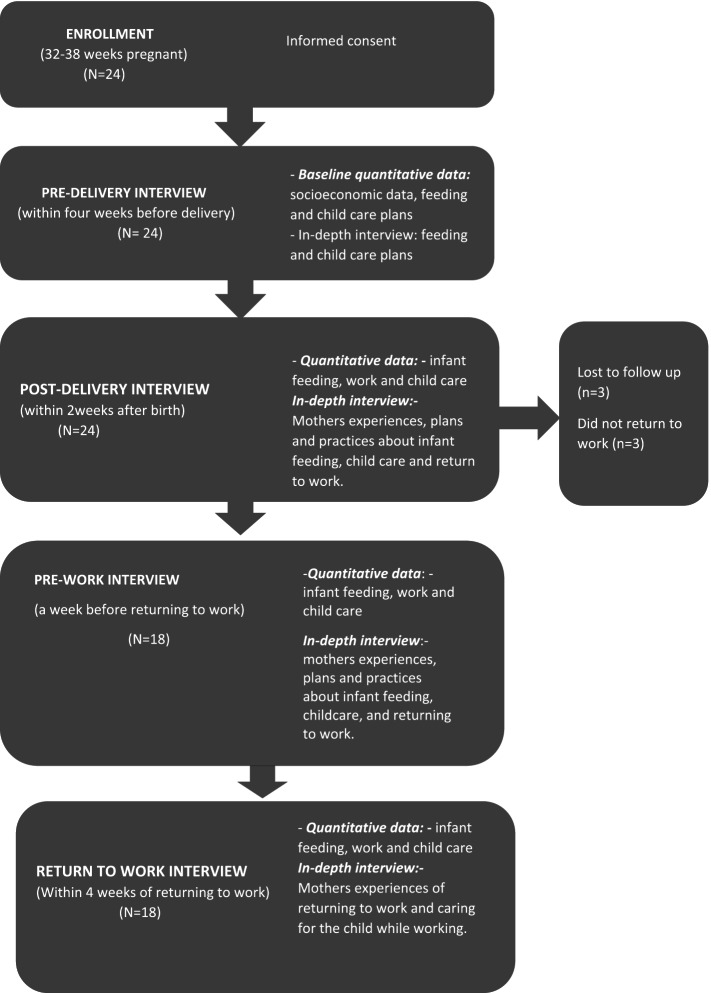
Cohort diagram

We conducted a series of interviews over the one-year follow up period which included both quantitative questionnaires and qualitative in-depth interviews (IDIs). The data was collected at different time points as follows: pre-delivery, within 2 weeks post-delivery, before and after returning to work, and when the baby was left in childcare (Fig. 1). The quantitative data included demographic data and food insecurity using the Household Food Insecurity Access Scale (HFIAS) [34]. During IDIs, participants were asked about their plans and experiences of applying for the CSG. All interviews were conducted in IsiZulu by experienced researchers at a time and location convenient to the participants and were audio recorded. Interviews were between 15 and 60 min in length. Data was collected from each participant by the same researcher throughout, allowing rapport and trust to build up between researchers and participants. Researchers maintained contact with the participants by telephone calls every two weeks throughout the study period to monitor participants’ progress and arrange interviews.

### Data analysis

IDIs were transcribed verbatim and translated into English. Framework analysis using NVIVO v12.3 was used to analyse qualitative data. During qualitative analysis the team of researchers first familiarised themselves with the data and coded a sample of the data to develop a coding framework. The coding framework was finalised during a series of meetings between researchers.

Quantitative data analysis used SPSSv26 and is presented as frequencies. Food insecurity was scored according to recommended HFIAS practices. HFIAS comprises nine questions where participants were asked about their access to food. If they indicated any lack of access to food, participants were asked to categorise it according to how often this happened (rarely, sometimes or often). Based on the answers, participants were categorised using the HFIAS system as no/mild food insecurity, moderate food insecurity, or severe food insecurity. Participants were considered severely food insecure if they reported any of the three most severe conditions such as running out of food, going to bed hungry or going a whole day and night without food, or if they *often* had to eat smaller meals or eat fewer meals in a day. Participants were designated moderately food insecure if they were eating a monotonous diet *often or sometimes*, or reducing the size of meals *rarely or sometimes*. Participants were designated as no or mild food insecurity if they had no restrictions to accessing food or if they only *sometimes* worry about accessing food [35].

## Results

Data was collected between July 2018 and August 2019. Twenty-four informal working women were enrolled in the cohort during pregnancy, and 87 interviews were conducted with the women over the study period. These included 24 interviews at pre-delivery, 24 post-delivery, 18 pre-work, 18 after return-to-work, and four baby-in-care interviews (Fig. 1). The median age of participating informal workers was 28.5 years (SD 4.7; IQR 25.0-30.7) at the pre-delivery interview.

Among 24 participants, 18 mothers returned to work, three mothers lost their jobs and did not return to work and three mothers were lost to follow up before returning to work. Most informal workers (10) returned to work within two months of the birth of the baby. Sociodemographic characteristics are shown in Table 1.

**Table 1:** Socio-demographic characteristics of participants

	All Women *N*=24
Age	
18-25 years	6
26-30 years	12
31-35 years	4
36 and over	2
Education	
Secondary school Grade 8-11	17
Completed school	7
Current relationship status/family situation	
Single	1
Married	1
In a relationship and living with partner	13
In a relationship and not living with partner	9
Number of other biological children	
First pregnancy (no other children)	4
1	11
2	5
3	4
Monthly income	
≤ R1000 (≤US$72)	6
R1000 -R3000 (US$72 - US$215)	16
≥ R3000 (≥US$215)	2
Received financial contribution from partner during pregnancy	22
Planned to take time off before childbirth	
Yes	4
No	9
Already stopped working at baseline	11

We found high rates of food insecurity at the pre-delivery interview that persisted until the return to work interview for some participants. Many informal workers reported moderate or severe food insecurity during the period before and after the birth of the baby (Table 2). However, mothers rarely discussed or mentioned that they sometimes had to miss meals.

**Table 2:** Food insecurity among participants at different time points

	Defined as	Pre-delivery (*N*=24)	Post-delivery (*N*=24)	Pre-work (*N*=18^a^)
No or mild food insecurity HFIAS score	Household members have access to sufficient nutritious food (food secure) or are sometimes limited in diversity or preferred choices (mild food insecurity).	6	10	10
Moderate food insecurity	Household members have access to lower quality food and are sometimes limited either in number and/or size of meals.	10	7	4
Severe food insecurity	Household members often had to limit number or size of meals and/or had no food +/- gone to bed hungry.	8	7	4

^a^Three mothers lost their jobs and did not return to work, three mothers were lost to follow-up before returning to work

*… He (baby) eats food now – porridge, and I sometimes buy Nespray for him, like two packets – he eats that. If it is finished, it is finished because I do not have support from anyone (M02_Did not return to work).*

The women in our study relied heavily on the CSG to pay for food and basic household services, but they experienced multiple challenges in applying and accessing the CSG for newborn babies. This increased their vulnerability and, in some cases, led to food insecurity and early return to work. The findings are presented according to four main themes: sources of income while not working before and after childbirth, the role of the CSG in supporting the household, delays in receiving the CSG, and the impact of such delays.

### Sources of income during leave from work before and after childbirth

Many women had stopped working in the last trimester of pregnancy, and relied mainly on the financial support from the father of the baby (19/24), and the CSG received for their older children (13/24) as sources of income while not working. The CSG was often the only consistent and reliable source of income during this period, because other income sources were insecure and varied according to circumstances, for example the father’s income or other financial commitments of family members. However, most women made plans to apply for the CSG as soon as the baby was born as an additional source of income.

*I hope I can register him for a child support grant because I cannot rely on his father because he is not supporting me even now. I will apply for a child support grant for him and then I will see when he is older if I can go back to (informal) work while (I am) looking for another job that will pay me better (M02_Baseline).*

### The importance of CSG in supporting the household

Even when she was working, the women’s income from informal work was often unpredictable, so the CSG was important as a reliable income source to provide for essentials. In particular, while the mother was not working before and after the birth of her child, the CSG received for older children was vital in providing for household and baby needs. However, delays in receiving the CSG meant that the CSG for the newborn baby was not available in the early weeks of the child’s life, and for first time mothers the CSG was not available until the application was complete, so they did not have support after the baby was born.

*I depend on the child support grant but I have not yet registered for her (new baby). I have the child support grant for the other 2 children … There are a lot of us here at home and we contribute R500 a month…so we can buy food. I am going to have a problem next month because I have to contribute R500 for [food]; perhaps I have to buy nappies…since I am not working it is tough; it is going to be tough (M16_Post-delivery).*

For some women, delays in receiving the CSG funds forced them to return to work early. The CSG money for older children was not enough for all the baby and household needs, leading to early return to work.

*It was the financial circumstances [that made me go back to work]. The child needs nappies. We also need money in the house. We were relying on the child support grant money but it is insufficient. There are too many things that are needed (M23_Return to work).*

### Delays in receiving CSG

At the end of the follow up period, ten participants had not applied for the CSG because of a variety of challenges (Table 3), and in one case because the mother was able to manage without the grant. A further four women had completed the application but had not yet received the grant and nine had received the grant. One mother was lost to follow-up before she applied for the grant. Among nine mothers who had received the grant, most had received it before the baby was 3months old (7), others waited for more than 6months (2). The four mothers who were still waiting for the grant had children aged between 2 and 11 months.

**Table 3:** Challenges in applying for the CSG reported by the participants

SASSA requirements for CSG application	Common Challenges	Process required	Participants experiences	Participant example
IDENTITY (ID) DOCUMENT for mother ID document and proof of marriage for married applicants Fathers ID for applicants with father’s name on child’s birth certificate	o Applicant has never had an ID o Missing or lost IDfor primary caregiver o Missing or lost ID for spouse/child’s father	First time ID applicants must be 16 years and above, and have a birth certificate, proof of address and must be accompanied by parent with their own ID book/smart card for the application. If applicant or spouse/child’s father has lost his ID, they have to go to Home affairs to apply for re-issuing of the document and receive the Temporary Identification Certificate (TIC). The application process takes 14 working days and the TIC is available during that time.	Two participants did not have an ID (M10 & M15) and one father had no ID (M05)	*No. I still have to apply for an identity document … then I can do a birth certificate for the baby and apply for a child support grant for her (M10_Post-delivery).* *No, I have not yet applied for it because her father lost his identity book. So we are still waiting for her father to get a replacement identity document and then he will go and apply with it (M05_pre-work).*
PROOF of INCOME	o Documents unavailable o Lack of knowledge about application and documents required o Lack of knowledge about application procedures for married applicants o Father does provide documents	Applicants must prove they earn less than R4000 per month for single applicants or R8000 per month for applicants who are married or in a relationship. This requires bank account statements with the application (3 months). If married or in a relationship with the father, the applicant needs to also provide father’s bank statements.		*I went to the Grant Office and they gave me forms which I filled out and took to the police station to get them stamped. I then returned the forms and they asked for my bank statement (M14_Pre-work).* *I have plans for it [applying for CSG] but I do not know if the application will succeed since I am married. They will ask about the child’s father because usually when you apply for a child support grant the assumption is that the child’s father is absent or does not support the child (M06_Post-delivery).*
PROOF OF EMPLOYMENT OR UNEMPLOYMENT LETTER from the Department of Labour for applicant and spouse/ father of the baby	o Incorrect employment status registered on the Department of Labour system	Applicants need to include pay slips with the application. Single caregivers: need to earn less than R48 000 per annum. Married caregivers or those in a relationship with the father: need to earn less than R96 000 per annum as a couple. If unemployed or informally employed, applicants need to include a proof of unemployment letter obtained from the Department of Labour.	Unable to provide proof of unemployment status despite being unemployed (M04 & M06)	*When I got to the Department of Labour I was told that where I work I am registered as a permanent employee … They gave me a letter stating same. They said I must take that letter back to SASSA … When I got there the official said to me “you are registered as a permanent employee and you earn a lot of money, so you are not eligible for a child support grant” … I asked her where I work. I wanted her to tell me the name of the company that I work for. She said I must go back to the Department of Labour because she was not going to be able to give me any information, except what was written on the letter…The official had also told me that the grant that I was receiving for the other two children will be stopped (M04_Pre-work).*
BIRTH CERTIFICATE for new baby *and* for older children who receive a grant	o Delayed issuing of birth certificate. o Home affairs systems unreliable o Applicant not South African o Extra traveling costs to retrieve birth certificates of older children	Applicants need to get a letter from the hospital confirming the birth of the child. This is taken to Home Affairs Department to apply for a birth certificate. In some instance home affairs offices are at the hospital.	Delayed birth certificate (M07, M22, M18, M02) M17 is not a South African citizen but the father of the child is South African. They both need to do DNA test to prove that they are parents before applying for birth certificate.	*Her birth certificate has not yet been issued. I was at [name of hospital] yesterday and they said I must wait 10 days and then go and collect it from Home Affairs (M21_Post-delivery).* *… they said you are not from this country. But father of the baby is from this country so you cannot [apply for birth certificate], you supposed to go to [name of hospital] to do Identity test [DNA test]. So identity test [DNA test] for the 3 of us is 2.2 [R2 200], father and child is 1.2 [R1 200] … So the father of the baby said he is looking for the money to go [name of hospital] to do identity test [DNA test] (M17_Return to work)*
SCHOOL ATTENDANCE CERTIFICATE for older children receiving a grant aged between 7 and 18 years old	o Time and travel costs to retrieve school attendance certificates of older children	Applicants must include school attendance certificates of all children under her care who are receiving the CSG as part of the new application.	Has to go to another province to get school certificate for older children (M21 & M24)	*Ok. I was going to apply for it at home [Eastern Cape]. The problem is that they wanted a letter. I have two children, right? The one that is at home goes to school. So now I will go home and ask for a letter from the school and then apply for a grant for the younger child (M24_Return to work).*

The application process as described by the women was long, time consuming, complex, and required a lot of paperwork (Table 3). Women reported that they had to go through various offices and departments to get forms filled in and approvals for the application, and they often had to carry the baby with them. These trips incurred costs that placed additional financial pressure on vulnerable women. In addition, several mothers required the father to take time off work to provide documents, leading to further delays, since this was outside of the mothers direct control.

*… It is such a stringent process. I have to go to the Department of Labour to look for a letter then go back to the hospital to sign; I have to get a letter from the hospital; then go to Home Affairs; it is so much work to go to SASSA; … It costs a lot of money to go to all these places plus I have to carry the baby with me … (M09_pre-work).*

Some women lacked understanding of the processes involved in the application and did not prepare the documents or get missing documents re-issued in preparation for applying for the CSG and some did not know how to go about starting the application process.

*No I do not even know where you register for that. Please tell me where do you go to register to apply for it? I am serious; I do not know where you apply for it (M08_Pre-work).*

### The effects of delays in the CSG application

Delays in the application process and receipt of CSG funds increased the vulnerability for women and their children. Women resorted to different coping strategies to address their lack of resources, these included borrowing money from family/neighbors and loan sharks, and others returned to work early.

*I could not stay home any longer. Furthermore, in my line of work if I do not work I do not get money because there is no one that is paying me a salary. If I want to earn money I have to work. So, if I was going to stay home longer I would not have had any income (M05_Return to work).*

For some of these women, returning to work meant stopping the CSG application process because taking time off work was unacceptable to employers, or time off work reduced the mother’s income.

*That thing takes time, and her birth certificate was delayed and I was about to return to work and would not have had time. Yeah, so if I had not returned to work I would be taking her to register [for CSG] (M07_Return to work).*

## Discussion

Cash transfer programmes have been implemented in many low- and middle- income countries globally, with wide-ranging benefits to education, nutrition, and health in poor communities [22, 24] and positive redistributive effects [25]. In South Africa the constitution guarantees the right to access to sufficient food, social security and assistance for people who cannot support themselves and their dependants [36]. The CSG is a cornerstone of government poverty relief interventions. This study adds to the literature about the CSG by highlighting the particular difficulties faced by informal workers, who are a large and vulnerable group with no maternity protection, in applying for the CSG and the impact this has on their daily lives. In addition, the prospective qualitative study design allowed participants to describe the challenges they experienced as they occurred over the follow-up period. Our findings echo the findings of several previous studies conducted since the inception of the CSG in 1998 [17, 18, 23]. The benefits of the CSG throughout childhood are well established, and the CSG is globally recognized as a hugely successful poverty relief programme [37, 38]. However, the application process is time-consuming, expensive and lengthy, and deprives mothers and children of the benefits of the CSG at a time when they are most needy and vulnerable. Despite ongoing and continued evidence of challenges women face in obtaining the grant timeously, our study shows these challenges persist currently, suggesting that no effective action has been taken. This study is particularly timeous given the rapid and effective systems implemented in South Africa to provide emergency support grants for individuals affected by COVID-19, which demonstrate clearly that mechanisms exist to address delays to CSG applications.

The CSG has several benefits to children and families throughout the years of childhood, but alleviating hunger and household food insecurity is the most crucial. However, despite these benefits there has been little or no improvement in the prevalence of stunting among South African children in the years since CSG implementation. Stunting remains a major public health problem despite high uptake and widespread coverage of the CSG [25]. Reasons for persistent high rates of stunting are multifactorial, and may include a poor or unsafe environment, poor quality of childcare, and poor access to health services. However, our study provides insights into other possible reasons for this from the perspective of informal workers, simply that the grant is received too late when a major opportunity for improving child nutrition has already passed, given that the largest benefits to child health are accrued during the early nutrition period when children are most vulnerable [23].

Women in our study described severe hardships during the period before and after the birth of a new baby when they were unable to work while also experiencing the financial pressure of providing for a newborn. Women with older children relied on the CSG as the only stable, reliable income for the family but women having their first baby were frequently left with no financial support. The effect of the delay in receiving the CSG for many of our participants was poverty, household food insecurity and an early return to work. Among informal workers in our study early return to work disrupted breastfeeding or led to mothers stopping breastfeeding altogether, with the need to buy formula adding to mothers’ financial pressures, as reported elsewhere [27]. In addition, early return to work reduced the time available to continue with the CSG application, which was therefore further delayed. Inadequate resources may also mean that mothers returning to work are unable to access good quality, safe childcare [30]. Further, it is likely that other essential family health needs are neglected due to lack of resources, for example clinic visits for postnatal care or infant vaccination, so that delays in accessing the CSG are likely to have wider implications for maternal and child health.

The early weeks of a child’s life are the foundation of a healthy future. Breastfeeding is crucially important for lifelong health and development, and spending time with your baby to breastfeed is a once in a lifetime opportunity. Unless the mother receives the support she needs to stay at home and continue to breastfeed, this opportunity will be missed with lifelong consequences for the child’s future achievements [39]. There is evidence that the greatest benefits of the CSG are shown among children whose carer received the grant while the child was still young [23, 39]. We suggest that providing the CSG immediately after birth would fill a crucial resource gap for informal working women and other low-income families, and could give these mothers the opportunity to breastfeed and provide for their family, giving the children the chance to thrive [18].

New systems required to achieve this are likely to have significant costs, but this should be seen in the context of the benefits. Improved health and cognitive development associated with good nutrition in early childhood will have long term economic benefits for the individual, for families and communities [23]. There will be immediate economic benefits for families who no longer have to undertake costly travel with a young baby to government offices to provide documentation. We argue that the economic and health benefits of improved systems for CSG applications would have long term economic as well as health benefits, that would outweigh any additional costs, in both the long and short term [23]. Further, we suggest that extending the current CSG into pregnancy to alleviate poverty also has proven benefits for pregnancy outcomes, leading to reduced maternal and child morbidity and mortality, and would also ensure that the grant is available immediately after delivery [22]. Many of the barriers to CSG application would be avoided if the application was processed during pregnancy when the woman does not have a small baby to care for, regardless of whether CSG payments start before or after delivery. The application process could be facilitated in the antenatal clinic by providing information, relevant documentation and even access to online resources to complete the application, which could be finalized at delivery when the baby’s birth certificate is issued.

Innovative technologies are already available to support the application process. During the COVID-19 epidemic the government introduced an unemployment grant. To roll this out quickly the South African Social Service Agency (SASSA) employed technology which allowed applicants to access this grant using platforms such as WhatsApp, SASSA website, SASSA email address and a toll-free number. Despite some challenges, this system proved to be effective, and approximately 9.7 million people accessed the special COVID-19 relief grant. We propose that SASSA expand this system to every CSG applicant to save time and money for mothers, and ensure that mothers and newborns receive the support they need.

## Conclusions

The CSG is a powerful tool for alleviating poverty and improving outcomes for the next generation of economically active South Africans but long delays deprive mothers and children of support at a crucial time. These delays are unnecessary since effective technologies exist to prevent them. We suggest that this is the time to harness new technologies to ensure that the CSG is available to mothers of newborns, and extended to pregnant women. This would provide support to the most vulnerable women and children when it is most needed, giving children a better start in life, with health and economic benefits that far outweigh the costs.

## Data Availability

All data, transcripts and study tools to support the findings of this study are available from the Centre for Rural Health and will be made available upon reasonable request from the principle investigator or corresponding author.

## Abbreviations

CT: Cash transfers
CSG: Child Support Grant
HFIAS: Household Food Insecurity Access Scale
ID: South African identity document
IDI: In-depth interview
SA: South Africa
SASSA: South African Social Service Agency

## References

[1] Ben Haman O (2019). Conditional and unconditional cash transfer programs: the recent experiences around the world. International Journal of Research and Innovation in Social Science.

[2] Molyneux M, Thomson M (2011). Cash transfers, gender equity and women’s empowerment in Peru, Ecuador and Bolivia. Gender & Development.

[3] Powell-Jackson T, Morrison J, Tiwari S, Neupane BD, Costello AM (2009). The experiences of districts in implementing a national incentive programme to promote safe delivery in Nepal. BMC health services research.

[4] Lim SS, Dandona L, Hoisington JA, James SL, Hogan MC, Gakidou E (2010). India’s Janani Suraksha Yojana, a conditional cash transfer programme to increase births in health facilities: an impact evaluation. The Lancet.

[5] Sosa-Rubí SG, Walker D, Serván E, Bautista-Arredondo S (2011). Learning effect of a conditional cash transfer programme on poor rural women’s selection of delivery care in Mexico. Health policy and planning.

[6] Paes-Sousa R, Santos LMP, Miazaki ÉS (2011). Effects of a conditional cash transfer programme on child nutrition in Brazil. Bulletin of the World Health Organization.

[7] Levy D, Ohls J (2010). Evaluation of Jamaica’s PATH conditional cash transfer programme. Journal of Development Effectiveness.

[8] Briaux J, Martin-Prevel Y, Carles S, Fortin S, Kameli Y, Adubra L, Renk A, Agboka Y, Romedenne M, Mukantambara F (2020). Evaluation of an unconditional cash transfer program targeting children’s first-1,000–days linear growth in rural Togo: A cluster-randomized controlled trial. PLoS medicine.

[9] Cherayi S, Jose JP (2016). Empowerment and social inclusion of Muslim women: Towards a new conceptual model. Journal of rural studies.

[10] Rawlings LB, Rubio GM (2005). Evaluating the impact of conditional cash transfer programs. The World Bank Research Observer.

[11] Prabhu KS, Sahay R, Roy R, Vinay C: Conditional cash transfer schemes for alleviating human poverty: Relevance for India. Discussion Paper, No 1 United Nations Development Programme (UNDP), India 2009.

[12] Fiszbein A, Schady NR: Conditional cash transfers: reducing present and future poverty: World Bank Publications; 2009.

[13] SASSA: BRANCH: STRATEGY AND BUSINESS DEVELOPMENT:TWELVETH STATISTICAL REPORT: PAYMENT SYSTEM MARCH 2021. 2021.

[14] Zembe-Mkabile W, Doherty T, Sanders D, Jackson D (2014). Child Support Grant access and receipt among 12-week-old infants in an urban township setting in South Africa. Global health action.

[15] Zembe-Mkabile W, Surender R, Sanders D, Jackson D, Doherty T (2015). The experience of cash transfers in alleviating childhood poverty in South Africa: mothers’ experiences of the Child Support Grant. Global public health.

[16] DSD, SASSA, UNICEF: The South African Child Support Grant Impact Assessment: Evidence from a survey of children, adolescents and their households. Pretoria: UNICEF South Africa 2012.

[17] Case A, Hosegood V, Lund F (2005). The reach and impact of Child Support Grants: evidence from KwaZulu-Natal. Development Southern Africa.

[18] Delany A, Ismail Z, Graham L, Ramkissoon Y: Review of the child support grant: Uses, implementation and obstacles. Johannesburg: Community Agency for Social Enquiry 2008.

[19] Stats S: Poverty trends in South Africa: An examination of absolute poverty between 2006 and 2015. Pretoria: statistics south Africa 2017.

[20] Zembe-Mkabile W, Surender R, Sanders D, Swart R, Ramokolo V, Wright G, Doherty T: ‘To be a woman is to make a plan’: a qualitative study exploring mothers’ experiences of the Child Support Grant in supporting children’s diets and nutrition in South Africa. BMJ open 2018, 8(4). 10.1136/bmjopen-2017-019376 PMC5922468 29691242

[21] Granlund S, Hochfeld T (2020). ‘That Child Support Grant Gives Me Powers’–Exploring Social and Relational Aspects of Cash Transfers in South Africa in Times of Livelihood Change. The Journal of Development Studies.

[22] Chersich M, Luchters S, Blaauw D, Scorgie F, Kern E, Van den Heever A, Rees H, Peach E, Kharadi S, Fonn S (2016). Safeguarding maternal and child health in South Africa by starting the Child Support Grant before birth: Design lessons from pregnancy support programmes in 27 countries. South African Medical Journal.

[23] Aguero J, Carter M, Woolard I: The impact of unconditional cash transfers on nutrition: The South African Child Support Grant. 2006.

[24] Coetzee M (2013). Finding the Benefits: Estimating the Impact of The S outh A frican Child Support Grant. South African Journal of Economics.

[25] Devereux S, Waidler J: Why does malnutrition persist in South Africa despite social grants. Food security South Africa working paper series 2017(001).

[26] Horwood C, Haskins L, Hinton R, Connolly C, Luthuli S, Rollins N (2021). Addressing the interaction between food insecurity, depression risk and informal work: findings of a cross-sectional survey among informal women workers with young children in South Africa. BMC Womens Health.

[27] Luthuli S, Haskins L, Mapumulo S, Rollins N, Horwood C (2020). ‘I decided to go back to work so I can afford to buy her formula’: a longitudinal mixed-methods study to explore how women in informal work balance the competing demands of infant feeding and working to provide for their family. BMC public health.

[28] Horwood C, Haskins L, Alfers L, Masango-Muzindutsi Z, Dobson R, Rollins N (2019). A descriptive study to explore working conditions and childcare practices among informal women workers in KwaZulu-Natal, South Africa: identifying opportunities to support childcare for mothers in informal work. BMC pediatrics.

[29] Leibbrandt M, Woolard I, Finn A, Argent J: Trends in South African income distribution and poverty since the fall of apartheid. 2010.

[30] Horwood C, Hinton R, Haskins L, Luthuli S, Mapumulo S, Rollins N (2021). ‘I can no longer do my work like how I used to’: a mixed methods longitudinal cohort study exploring how informal working mothers balance the requirements of livelihood and safe childcare in South Africa. BMC Women’s Health.

[31] South African population census 2011 [http://www.statssa.gov.za/?page_id=993&id=ethekwini-municipality]

[32] Jama NA, Wilford A, Masango Z, Haskins L, Coutsoudis A, Spies L, Horwood C (2017). Enablers and barriers to success among mothers planning to exclusively breastfeed for six months: a qualitative prospective cohort study in KwaZulu-Natal, South Africa. International breastfeeding journal.

[33] Horwood C, Haskins L, Luthuli S, McKerrow N (2019). Communication between mothers and health workers is important for quality of newborn care: a qualitative study in neonatal units in district hospitals in South Africa. BMC pediatrics.

[34] Castell GS, Rodrigo CP, de la Cruz JN, Bartrina JA (2015). Household food insecurity access scale (HFIAS). Nutricion hospitalaria.

[35] Coates J, Swindale A, Bilinsky P: Household Food Insecurity Access Scale (HFIAS) for measurement of food access: indicator guide: version 3. 2007.

[36] The Constitution of the Republic of South Africa. In.; 1996.

[37] DSD SASSA, UNICEF (2016). Removing barriers to accessing Child Grants: Progress in reducing exclusion from South Africa’s Child Support Grant.

[38] Patel L, Knijn T, Van Wel F: Child support grants in South Africa: a pathway to women’s empowerment and child well-being?Journal of social Policy 2015, 44(2):377-397.

[39] Victora CG, Bahl R, Barros AJ, França GV, Horton S, Krasevec J, Murch S, Sankar MJ, Walker N, Rollins NC (2016). Breastfeeding in the 21st century: epidemiology, mechanisms, and lifelong effect. The Lancet.

